# Layer-specific quantitative analysis of accommodation in the human lens using long-range OCT and machine learning

**DOI:** 10.1186/s40662-026-00517-w

**Published:** 2026-11-01

**Authors:** Keerthana Soman, Muhammad Usman, Raul Urriza-Arpal, Grzegorz Gondek, Karol Karnowski, Jacek Ruminski, Ireneusz Grulkowski

**Affiliations:** 1 Institute of Physics, Faculty of Physics, Astronomy and Informatics, Nicolaus Copernicus University in Toruń, Grudziądzka 5, 87-100 Toruń, Poland; 2 Institute of Advanced Studies, Nicolaus Copernicus University, Toruń, Poland; 3 Department of Biomedical Engineering, Faculty of Electronics, Telecommunications and Informatics, Gdansk University of Technology, Gdansk, Poland; 4 Institute of Physical Chemistry, Polish Academy of Sciences, Warsaw, Poland; 5 International Center for Translational Eye Research, Polish Academy of Sciences, Warsaw, Poland

**Keywords:** Accommodation, Optical coherence tomography, Optical signal discontinuity zones, Machine learning, Ocular biometry

## Abstract

**Background:**

The crystalline lens undergoes complex deformations during accommodation. However, in vivo characterization of the accommodation-induced dynamics of the internal lens structures has been hindered by limited imaging depth and by the difficulty of segmenting these subtle features. In this study, long-range optical coherence tomography (OCT) was used to visualize and measure the morphology of the accommodating eye, including the cornea, entire lens, nucleus, and optical signal discontinuity (OSD) zones (C1–C4) in the lens cortex.

**Methods:**

Sixty healthy young adults (mean age 26 ± 7 years) were imaged with a commercial long-range OCT system at four accommodative demands (0, 2, 4, and 6 D) using a modified Badal unit. Machine learning-assisted segmentation of the corneal and lens interfaces enabled correction of the OCT images for light refraction. The central densitometric depth profile was used to identify and manually delineate the anterior and posterior OSD zones in the crystalline lens cortex. The analysis of accommodation-related changes in eye geometry included intraocular distance, OSD-zone thickness, and surface curvature.

**Results:**

Accommodation induced significant thickening and curvature changes in both the whole lens and its nucleus. Zonal analysis revealed localized changes within the cortex, with the inner C4 zones showing the largest accommodative thinning and contributing to lens deformation, whereas the peripheral cortical zones exhibited minimal changes. These internal lens deformations were accompanied by typical biometric responses of the eye to accommodation, including reduced anterior chamber and vitreous chamber depths across demands, and a slight increase in ocular axial length at lower accommodative demands.

**Conclusions:**

This study provides a map of lenticular deformation during accommodation, identifying the cortical C4 zones and the lens nucleus as the primary deformation compartments. In contrast, the peripheral cortex remains relatively passive. These findings demonstrate that accommodation cannot be fully described using global lens metrics alone. Therefore, biomechanical and optical models should incorporate cortical and nuclear behavior instead of treating the lens as a homogeneous structure. The quantitative data presented here extend our understanding of the OSD zones and establish a baseline for future investigations of age-related and pathological alterations in accommodation and lens structures.

**Supplementary Information:**

The online version contains supplementary material available at https://doi.org/10.1186/s40662-026-00517-w.

## Background

The eye dynamically adjusts its focal power in response to retinal defocus. This accommodation process is primarily mediated by ciliary muscle contraction and zonular relaxation, which act together to alter the shape and thickness of the crystalline lens [1]. As the lens thickens, its anterior and posterior surfaces become more steeply curved, increasing the optical power of the eye to the level required for near vision [2–6]. Understanding the mechanisms governing these dynamic changes is essential for explaining the physiology of presbyopia, optimizing intraocular lens design, and diagnosing accommodative dysfunction. Although accommodation has been extensively studied, most research has focused on ocular biometric parameters and may not fully capture the complex internal reorganization of the crystalline lens underlying accommodative functions [5, 7–9].

The crystalline lens, the key component involved in accommodation, consists of distinct regions, including the cortex and the nucleus, a differentiation first described by Johann Gottfried Zinn in the eighteenth century [10]. Accommodation-induced changes occur mostly within the nucleus, a region composed of densely packed, highly ordered lens fiber cells filled with crystallin proteins [4, 11]. Very high concentrations of soluble crystallin proteins and their precise packing are fundamental for lens transparency [12]. Spatial variations in protein concentration and water content that develop during lifelong lens growth give rise to a gradient refractive index (GRIN), which further optimizes the optical quality of the eye [13, 14].

Fine perturbations associated with microheterogeneities in protein and water distribution cause small deviations from the otherwise smooth GRIN profile, which can be visualized as zones of optical discontinuity [12–15]. These subtle microstructural variations are observed using light-based imaging modalities [15–17]. Cross-sectional optical images of the crystalline lens reveal characteristic light and dark regions, referred to as optical signal discontinuity (OSD) zones, which serve as markers of the internal structure and organization of the lens [15, 17]. Based on Scheimpflug and slit-lamp imaging, the Oxford clinical cataract classification and grading system defines four cortical zones (C1–C4, including the subzones C1α and C1β) and the nucleus, which together reflect the layered growth pattern of the lens [18]. However, segmentation of the cortical C4 zone is usually challenging. In earlier structural work, the nucleus was defined as the area between the C3 zones while combining C1–C3 as the cortex, limiting insights into potential differences between the central and peripheral cortical layers [4]. More recently, swept-source optical coherence tomography (SS-OCT)-based studies have extended this zonal framework by visualizing all cortical zones and quantifying age-related changes in thickness and transparency within individual zones [17]. These findings highlight the sensitivity of OCT for resolving fine internal structures and capturing zone-specific patterns.

In contrast to these age-related investigations, studies on accommodation have not provided a complete, depth-resolved characterization of all OSD zones [7–9]. Conventional anterior segment OCT has been widely used to assess accommodative changes in lens geometry, but its restricted imaging range and reduced sensitivity at greater depths preclude simultaneous visualization of the entire ocular axis and evaluation of the internal lens structure [7, 19–21]. Consequently, the relationship between global biometric changes and localized structural remodeling of the crystalline lens during accommodation remains poorly understood.

In this study, we employed long-range SS-OCT combined with machine learning-assisted segmentation to enable whole-eye imaging during accommodation with sufficient contrast to resolve individual OSD zones. Our aim was to quantify the distribution of accommodative changes in the eye, with a specific focus on the structural regions of the lens, and to determine which zones contribute most to lens deformation. By integrating measurements of intraocular distance, curvature, and zonal thickness, we sought to provide a clinically interpretable, depth-resolved description of accommodative behavior.

## Methods

This prospective study was conducted in accordance with the tenets of the Declaration of Helsinki and was approved by the Bioethics Committee at the Nicolaus Copernicus University (KB 171/2024). Written informed consent was obtained from all participants before enrollment.

Sixty healthy young adults (mean age 26 ± 7 years; range 18–50 years; 38 males, 22 females) were recruited. Their mean spherical refractive error was 0.50 ± 0.76 D and their mean cylindrical refractive error was 0.13 ± 0.57 D. For imaging, one eye per participant was randomly selected (coin toss) to avoid bias and ensure uniform sampling across participants. The exclusion criteria included clinical signs of presbyopia, a history of ocular disease (excluding refractive error), and absolute refractive errors greater than 3.5 D. One participant with a history of corneal refractive surgery (laser-assisted in situ keratomileusis) was included in the final analysis because the eye met all other inclusion criteria.

### Imaging system

To capture the complete intraocular architecture during dynamic accommodation, a commercial long-range SS-OCT system (Atria, Thorlabs Inc.) was integrated with a custom Badal optometer. SS-OCT was performed using a microelectromechanical systems tunable vertical-cavity surface-emitting laser with a central wavelength of 1060 nm. The optical power incident on the cornea was 0.73 mW, which is below the exposure limits of safety standards [22]. Operating at 60,000 A-scans per second, the system achieved an axial imaging range of 40.2 mm and an axial resolution of 11 μm. This extended range enables full-length biometry in a single acquisition.

**Fig. 1:**
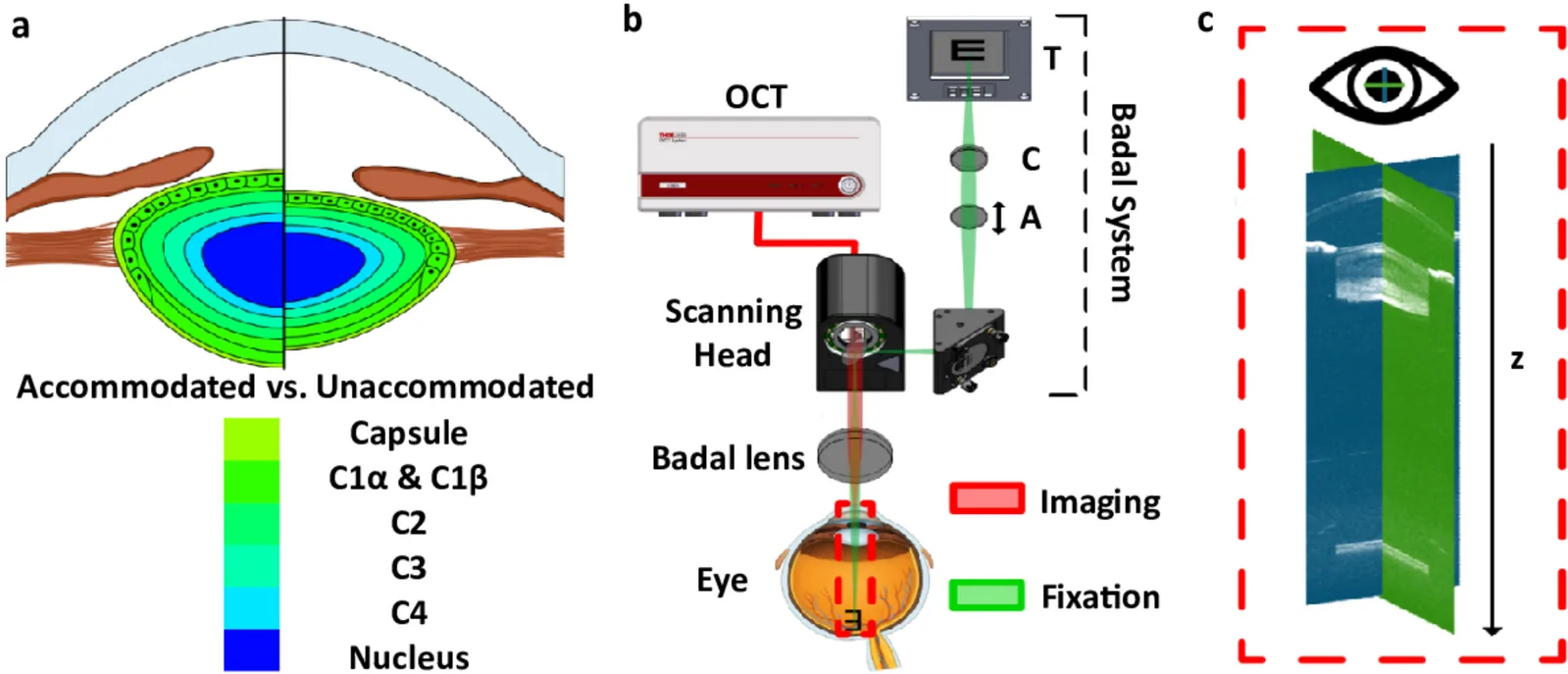
Lens optical signal discontinuity (OSD) zones and the long-range optical coherence tomography (OCT) imaging setup. **a** Schematic diagram of the crystalline lens during accommodation with the OSD zones labeled according to the Oxford nomenclature [18]; **b** Sample arm of the swept-source OCT (SS-OCT) system integrated with modified Badal unit [23]. A, auxiliary lens; C, collimating lens; T, target display; **c** Quasi simultaneous scanning of the eye enabling acquisition of cross-sectional images in two cardinal meridians (horizontal and vertical)

The Badal setup (Fig. 1b) enabled precise control and maintenance of accommodative demand. It consists of a high-resolution target display (LS029B3SX04; Sharp Corp.; T in Fig. 1b) positioned at the front focal plane of the collimating lens (f = 125 mm; Thorlabs; AC254-125-A-ML; C in Fig. 1b), followed by an auxiliary lens (f = 200 mm; Thorlabs; AC254-200-A-ML; A in Fig. 1b) mounted on a movable rail [8, 23]. The OCT objective lens served as a Badal lens (f = 110 mm; Thorlabs; OCT-LK5-BB; Fig. 1b). This configuration provided a stimulus range from −9.5 D to +4.6 D, with a linear displacement of 13.1 mm per 1 D of demand. This allowed for both neutralization of individual spherical refractive errors and systematic mapping of the ocular response across increasing accommodative steps. Changes in accommodative demand did not introduce angular magnification, so the apparent size of the fixation target remained constant throughout the stimulus range.

### Imaging protocol

The participants were seated with their heads stabilized using a chin and forehead rest. To ensure a natural physiological response, no pharmacological mydriatic agents were used. The baseline accommodative state (0 D demand) was determined subjectively by adjusting the auxiliary lens until the participant reported that the fixation target was in sharpest focus.

After baseline determination, the distance between the Badal lens and the auxiliary lens was adjusted systematically to induce accommodative efforts of 2, 4, and 6 D, and was then returned to 0 D to capture both accommodation and disaccommodation dynamics. The SS-OCT system was aligned with the optical axis of the participant’s eye, using the corneal apex reflection as a reference and centering the eye on the intersection of the two orthogonal meridians.

Data acquisition was initiated only after the participant verbally confirmed stable fixation on the target at each demand level. To account for potential asymmetry in accommodative response, quasi-simultaneous imaging of two perpendicular meridians (Fig. 1c) was performed within a single acquisition sequence, allowing accommodative changes in the shape of the ocular surfaces to be compared between the two meridians. Each acquisition resulted in two B-scans consisting of 1400 A-scans covering a lateral field of view of approximately 10 mm. At this scan rate, the total acquisition time per frame was approximately 80 ms, minimizing the impact of microsaccades and ensuring high-fidelity cross-sectional full-eye-length images. The initial data processing and hardware control were managed using a custom-made LabVIEW interface (National Instruments Corp.).

### Data post-processing

#### Machine learning model

**Fig. 2:**
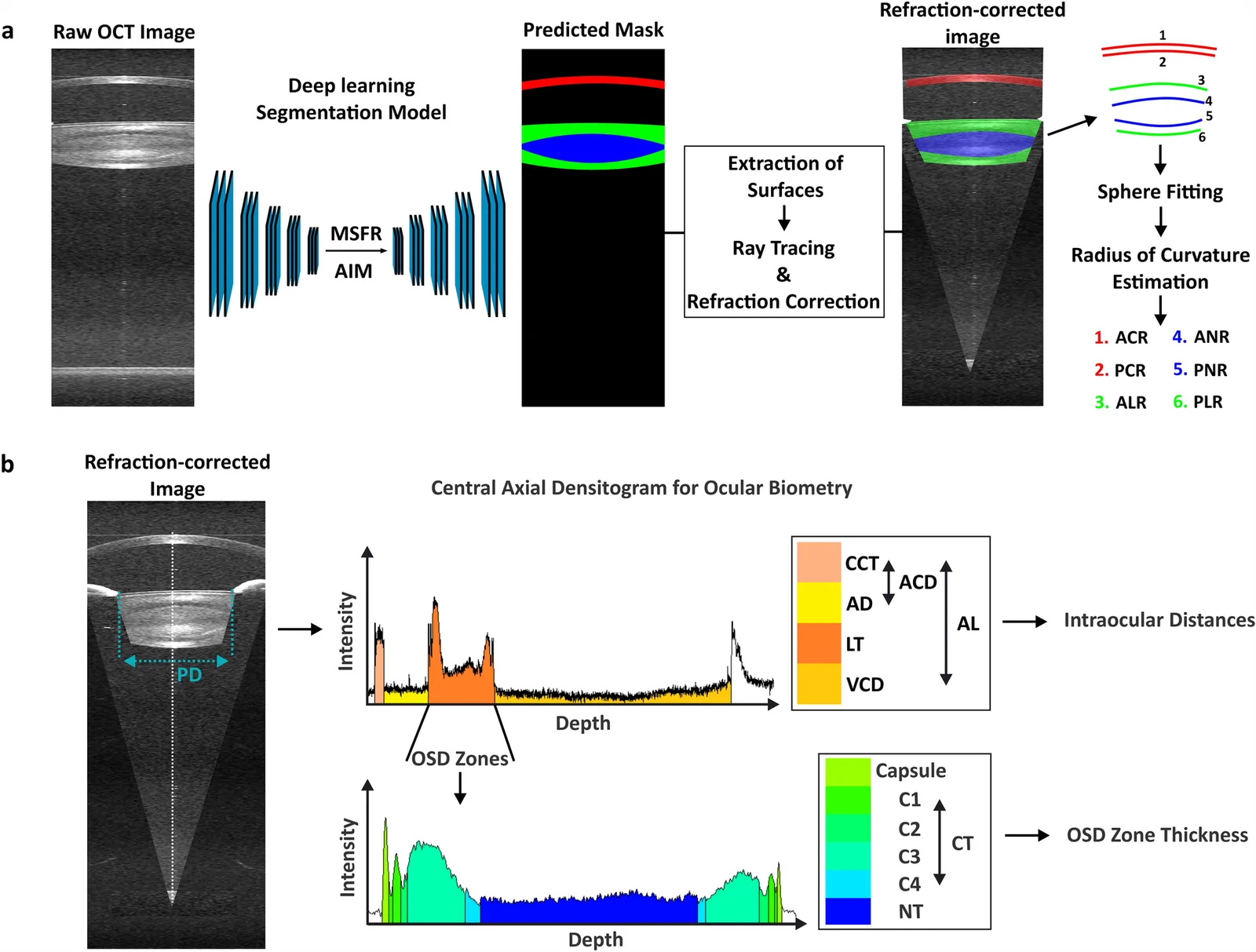
Schematic overview of the full-eye optical coherence tomography (OCT) analysis workflow. **a** Deep learning pipeline for training a segmentation model using annotated OCT images. The network included an encoder, a multiscale feature refinement module, and an attention-based feature integration module (AIM). The trained model generated pixel-wise masks of the ocular interfaces, whose boundaries were extracted for subsequent curvature analysis. **b** In a separate workflow, the refraction-corrected OCT images were used to estimate thickness and perform biometric measurements. ACD, anterior chamber depth; ACR, anterior corneal radius; AD, aqueous depth; AL, axial length; ALR, anterior lens radius; ANR, anterior nuclear radius; CT, cortical thickness; CCT, central corneal thickness; C1–C4, representing anterior and posterior optical signal discontinuity (OSD) zones; LT, lens thickness; NT, nuclear thickness; PCR, posterior corneal radius; PD, pupil diameter; PLR, posterior lens radius; PNR, posterior nuclear radius; VCD, vitreous chamber depth

The semantic segmentation of full-eye-length OCT images was performed using a deep learning architecture based on the U-Net framework [24]. This architecture comprised three components: a pre-trained residual network (ResNet-50) as an encoder for robust feature extraction, a multiscale feature refinement module at the bottleneck to incorporate contextual information, and an attention-based feature integration module to improve feature fusion between the encoder and decoder (Fig. 2a).

The model was evaluated both quantitatively and qualitatively on an unseen test dataset (536 images) and achieved strong segmentation performance, with a mean intersection over union (IoU) of 0.936 and high recall (0.974) and specificity (0.995). These results indicated a good agreement between the predicted and reference masks, with little over-segmentation and few missed boundary pixels. The high per-class IoU values for the cornea, lens, and nucleus (0.912, 0.913, and 0.928, respectively) further suggested reliable delineation of these main ocular structures in the evaluated dataset [24]. The final output provided a dense pixel-wise segmentation of the cornea, lens cortex, and lens nucleus surfaces, which were subsequently used for refraction correction and biometric calculations.

### Thickness measurement

Thickness was derived from the optical distances between surface boundaries along the central A-scan, which was extracted from the cross-sectional OCT image (Fig. 2b). An OCT-based densitogram was obtained from the averaged central A-scan profile and used to identify the ocular surfaces, from which the intraocular distances were derived (central corneal thickness [CCT], aqueous depth [AD], anterior chamber depth [ACD], lens thickness [LT], vitreous chamber depth [VCD], and axial length [AL]). The OSD zone boundaries were manually delineated by locating peaks and troughs in the densitometric profile and were subsequently verified against the corresponding B-scan to ensure spatial consistency with visible anatomical structures [17]. The thicknesses of the OSD zones C1–C4 were combined to obtain the cortical thickness (CT). Additionally, the innermost zone forming the nucleus was defined as nuclear thickness (NT) (Fig. 2b). The intra-observer reliability of the manually segmented OSD zones was assessed by computing within-session repeatability and inter-session reproducibility (coefficient of variation and intraclass correlation coefficient), which demonstrated good-to-excellent stability for most outer and intermediate zones at both 0 D and 6 D (see Table S1 in Additional file 1).

### Radius of curvature measurement

The machine learning model predicted segmentation masks for the cornea, lens, and nucleus, from which the corresponding surface curves were extracted for subsequent post-processing (Fig. 2a). These surface curves were then fed into a ray-tracing refraction-correction algorithm that treated the eye as a layered structure. Each A-scan was regarded as a ray propagating inside the eye, with the z-positions of the segmented interfaces corresponding to the optical distances between the boundaries. The custom-designed algorithm used Snell’s law to determine the direction of light propagation and recover the true geometric distances and the shapes of the ocular surfaces. The images were corrected for light refraction using medium-specific refractive indices along the ocular axis. We assumed refractive indices of 1.0000 (air), 1.3665 (cornea), 1.3266 (aqueous humor), 1.3749 (anterior cortex), 1.3947 (nucleus), 1.3749 (posterior cortex), and 1.3270 (vitreous humor) [17, 25]. These values were derived from the wavelength-dependent dispersion model of Atchison and Smith and evaluated at the central wavelength of the SS-OCT system [25].

In the final step, the refraction-corrected surfaces were fitted with a sphere over the central 3-mm zone, and the radius of the best-fit sphere was taken as the surface radius of curvature. The radii of curvature determined were the anterior and posterior corneal radii (ACR, PCR), the anterior and posterior nuclear radii (ANR, posterior nuclear radius [PNR]), and the anterior and posterior lens radii (ALR, posterior lens radius [PLR]). The pupil diameter (PD), defined as the distance between the visible pupillary margins in each scan, was measured manually from the refraction-corrected image (Fig. 2b).

Biometric measurements were performed for the four accommodative states. For each, the thickness values from horizontal and vertical scans, as well as from accommodation and disaccommodation sequences, were averaged to obtain a single representative value per state. When direction- or meridian-specific effects were analyzed, the corresponding measurements were treated separately rather than averaged. Changes in each measured parameter relative to the baseline (relaxed, 0 D) state were reported for the 2 D vs. 0 D, 4 D vs. 0 D, and 6 D vs. 0 D demand pairs. Because the absolute accommodative changes across several parameters were very small in physical units (mm), we also expressed these data as percentage changes from baseline.

### Statistical analysis

Data analyses were performed using Microsoft Excel 2013 (Professional Plus; Microsoft Corp., Redmond, WA, USA) and MATLAB (MathWorks Inc., Natick, MA, USA). The distribution of each variable was assessed using the Shapiro–Wilk test (*P* > 0.05, indicating no significant deviation from normality).

Paired comparisons between baseline (0 D) and accommodative demands (2, 4, or 6 D) were performed using paired t-tests for normally distributed data and Wilcoxon signed-rank tests for non-normal data. The Bonferroni correction was applied to adjust for multiple comparisons. For each parameter, subject-specific linear regression slopes across accommodative demands (rate of change per diopter) were calculated and tested against zero using one-sample t-tests or Wilcoxon tests, as appropriate.

Group comparisons were performed for sex, laterality, meridian, and accommodation direction. For normally distributed data, paired t-tests were used for the meridian and accommodation-direction comparisons, and Welch’s t-test for the sex and laterality comparisons, to accommodate unequal variances. For non-normal data, Wilcoxon signed-rank tests and Mann–Whitney U tests were used for the same comparisons, respectively. For each parameter, group differences in slopes (change per diopter) were tested to identify possible variation in accommodative dynamics. Structural differences were then evaluated in two steps: (1) baseline values at 0 D were compared to identify offsets, and (2) these offsets were checked for directional consistency across higher demands (2, 4, and 6 D) to determine whether one group showed systematically larger or smaller values across the full demand range. Parameters were classified as showing a structural difference only when baseline and across-demand comparisons supported a consistent directional difference.

In addition to these univariate analyses, we fitted a linear mixed-effects model for each biometric parameter and lens layer thickness, with participant as a random factor and accommodative demand, orientation, direction, sex, and eye as fixed factors. This accounted for the multilevel, multifactor structure of the data and for within-subject correlations, allowing baseline values at 0 D and changes across demands to be compared between groups.

All tests were two-tailed with a significance level of α = 0.05. Effect sizes were reported as Hedges’ g for parametric comparisons (t-tests) and rank-biserial correlations for non-parametric tests (Wilcoxon and Mann–Whitney), and 95% confidence intervals (CIs) were calculated for all main effects to quantify both the magnitude and precision of the estimated differences.

### Data visualization

Morphological parameters obtained from the processed OCT data were plotted against accommodative demand. The mean changes in the thickness and curvature with accommodation are summarized in the tables, and key plots are presented in the following sections. Distributions were visualized using raincloud plots, which combined smoothed density (cloud), individual data points (rain), and summary statistics (mean and standard deviation) for each accommodative demand. In these plots, the x-axis indicates the stimulus demand (0, 2, 4, and 6 D), the y-axis shows the change in thickness or curvature from the 0 D baseline, and the colored cloud depicts how frequently different values occurred across the eyes. The short black horizontal bar marks the mean and the vertical bar indicates the standard deviation for that condition.

Heatmaps summarize pairwise comparisons of layer-specific thickness and curvature changes between 0 D and 2, 4, and 6 D. The heatmap of Bonferroni-corrected *P* values used a relative color scale that was rescaled separately for each subgroup, so that the darkest red corresponded to the smallest *P* value and the coolest color to the largest *P* value within that subgroup. Effect sizes were visualized using a color scale truncated to the range −1 to +1; values with |effect size|> 1 were plotted at the scale limits. Whereas *P* values indicate whether the observed differences are unlikely to have arisen by chance, the effect size quantifies the magnitude and direction of these differences, helping distinguish statistically significant but trivial effects from changes that are both statistically and practically meaningful.

## Results

**Fig. 3:**
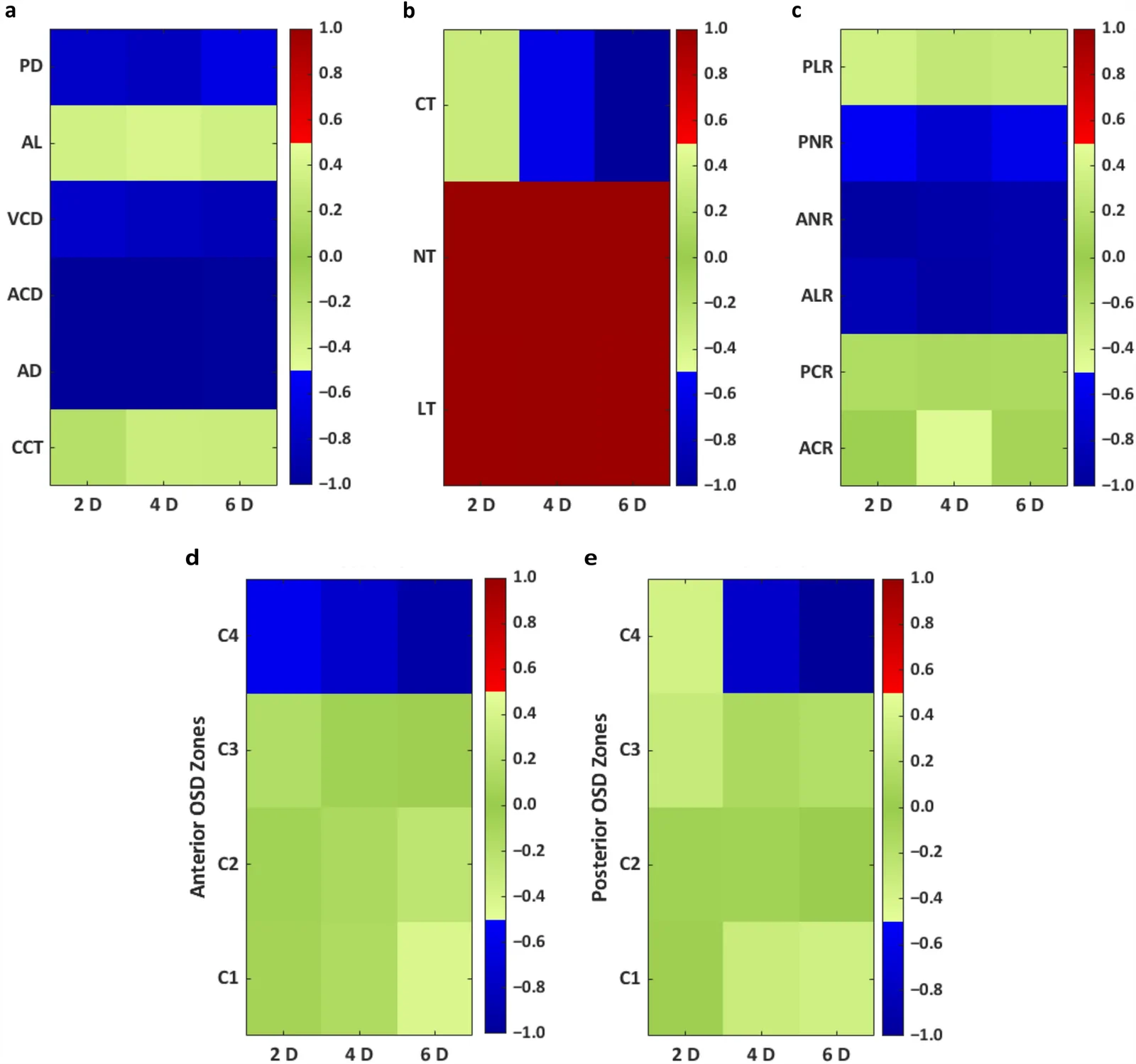
Combined effect size heatmaps for the parameters describing accommodation-induced changes. **a** Intraocular distances and pupil diameter (PD) across accommodative demands. **b** Thickness of the crystalline lens and its anatomical components; **c** Radii of curvature of the ocular surfaces; **d** Thicknesses of anterior cortex optical signal discontinuity (OSD) zones C1–C4; **e** Thicknesses of posterior cortex OSD zones C1–C4. ACD, anterior chamber depth; ACR, anterior corneal radius; AD, aqueous depth; AL, axial length; ALR, anterior lens radius; ANR, anterior nuclear radius; CCT, central corneal thickness; CT, cortical thickness; LT, lens thickness; NT, nuclear thickness; PCR, posterior corneal radius; PLR, posterior lens radius; PNR, posterior nuclear radius; VCD, vitreous chamber depth

For comparison across demands, we combined the effect size maps for all measured parameters, as shown in Fig. 3. The full set of pairwise comparisons between the baseline (0 D) and accommodative demand, expressed by the magnitude of change and P values at each demand and layer, is provided in Tables S2, S4, S6, and S8, and Fig. S1 [see Additional file 2].

In addition to the absolute changes presented here, we examined the same parameters as percentage differences from the baseline to facilitate the interpretation of small accommodative shifts. The group-mean percentage differences for all biometric, lenticular, and curvature parameters at 2, 4, and 6 D are provided in Tables S3, S5, S7, and S9 [see Additional file 2].

### Changes in the intraocular distances and PD

**Fig. 4:**
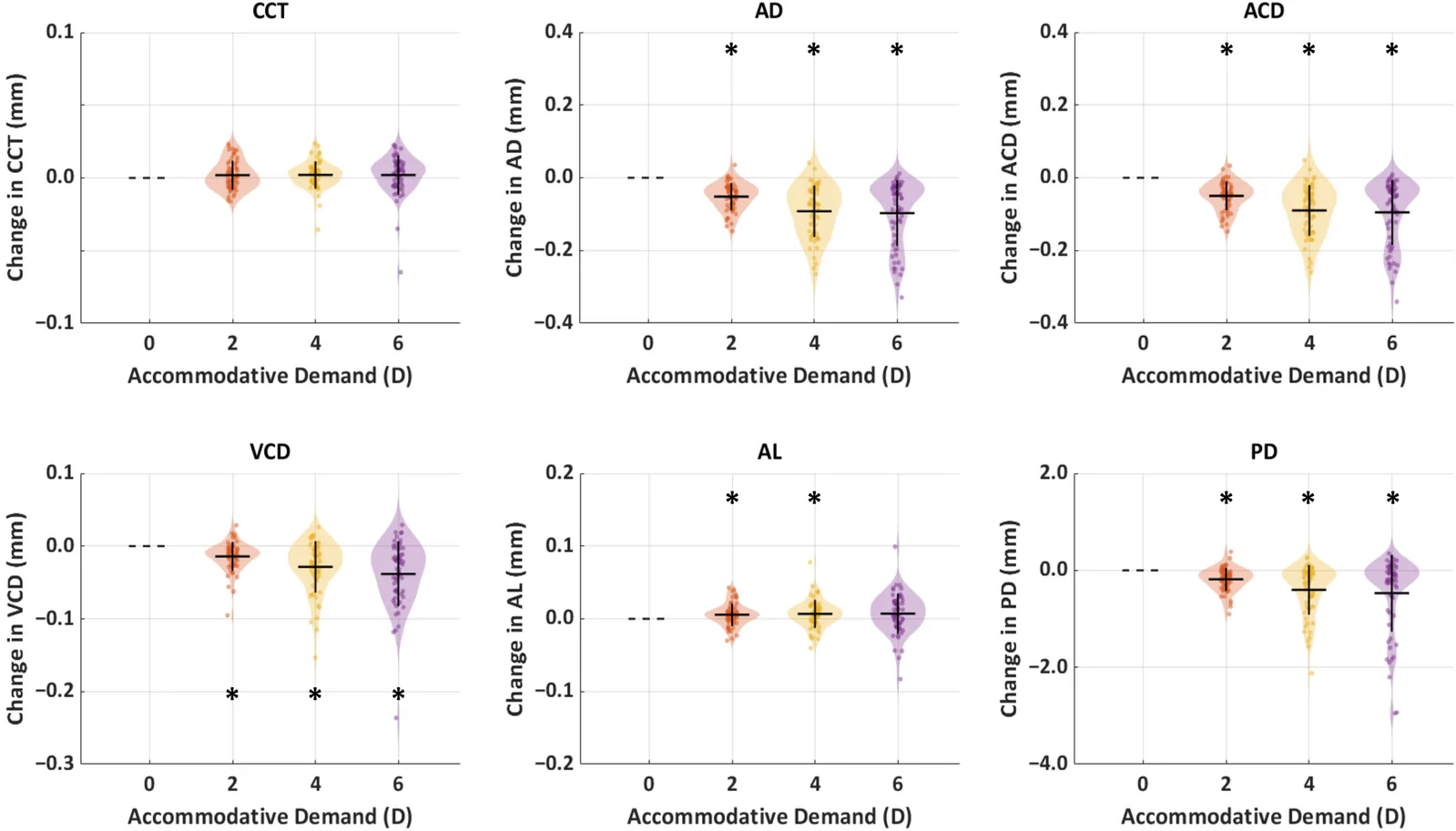
Raincloud plots of accommodation-related changes in intraocular distances and pupil diameter (PD). Asterisk indicates statistical significance with α ≤ 0.05. Each panel shows the distribution of change from the 0 D baseline at 2 D, 4 D, and 6 D for central corneal thickness (CCT), aqueous depth (AD), anterior chamber depth (ACD), vitreous chamber depth (VCD), axial length (AL) and PD

Intraocular distances showed systematic demand-dependent changes during accommodation (Fig. 4). After the Bonferroni correction, the CCT changes did not remain statistically significant at any accommodative demand. Because the changes are well below the axial resolution of the OCT system (11 μm), the observed differences likely reflect variability within its measurement precision rather than true accommodative remodeling. In contrast, AD and ACD decreased progressively with demand (*P* < 0.001); effect sizes ranged from −1.38 to −0.99 (95% CIs entirely below 0). VCD also shortened with accommodation, showing a similar demand-dependent decrease (*P* < 0.001; effect sizes: −0.85 to −0.76; 95% CIs entirely below 0). AL showed small positive changes that were significant at lower demands (2 D and 4 D; *P* < 0.05) but did not remain significant at 6 D after correction (*P* = 0.059), indicating a borderline effect that does not persist across the full accommodative range. Expressed per diopter, AD and VCD changed by −16.58 µm/D and −6.46 µm/D, respectively, whereas AL increased by about 1.12 µm/D and CCT showed rates close to 0 [see Table S2 in Additional file 2]. Percentage change analysis showed that all five global biometric parameters (CCT, VCD, AL, AD, and ACD) shifted by less than 3.5% across the full accommodative range [see Table S3 in Additional file 2].

A significant decrease in PD (−81.66 μm/D) was also observed with increasing demand (effect sizes: −0.81 to −0.62; 95% CIs well below 0). This was the largest relative change of any biometric parameter, reaching approximately 7.3% at 6 D.

The plots in Fig. 4 show the distribution of individual changes from the 0 D baseline at 2, 4, and 6 D for all intraocular distances and PD. For AD, ACD, and VCD, the distributions shifted to more negative values with increasing accommodative demand, whereas AL and CCT remained closer to zero. The PD showed progressively larger negative changes across demand. The heatmaps in Fig. 3a show the corresponding effect sizes for each outcome measure at 2, 4, and 6 D. Warmer colors indicate more positive effect sizes and cooler colors more negative effect sizes on a scale from approximately −1.4 to 0.4 (large negative effects for AD, ACD, VCD, and PD, and small positive effects for AL). In the P value panel, warmer colors represent lower and cooler colors higher P values [see Fig. S1a in Additional file 2].

### Changes in the thickness of the lens and its main components

**Fig. 5:**
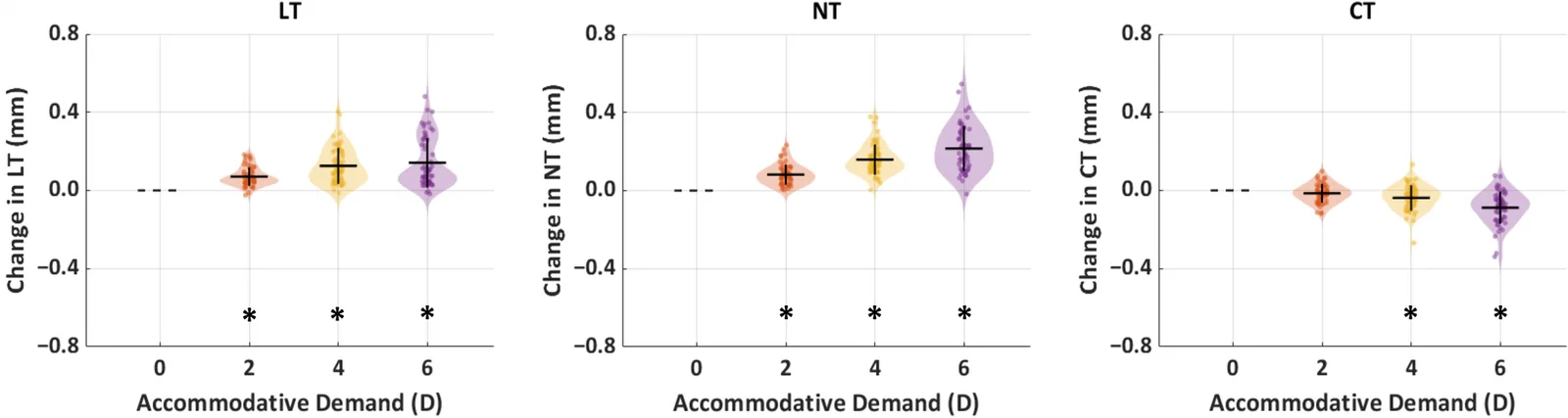
Raincloud plots of accommodation-related thickness changes in the crystalline lens and its components. Asterisk indicates statistical significance with α ≤ 0.05. Each panel shows the distribution of change from the 0 D baseline at 2 D, 4 D, and 6 D for lens thickness (LT), nuclear thickness (NT) and cortical thickness (CT)

The thickness of the entire lens and its components changed systematically with increasing accommodative demand (Fig. 5). LT increased significantly and almost linearly with accommodative demand (*P* < 0.001; effect sizes close to 1; 95% CIs entirely above 0). The nuclear region exhibited an even larger absolute increase in thickness (*P* < 0.001, effect size ≥ 1; 95% CIs entirely above 0). In contrast, the combined anterior and posterior CT decreased with increasing demand; this reduction was not significant at 2 D after Bonferroni correction (*P* = 0.056) but was significant at 4 D and 6 D (*P* < 0.001, effect size ≥ 0.61; 95% CIs entirely below 0), indicating thinning of the cortical layers as the nucleus thickened. Whole lens and NT increased at rates of approximately 24 μm/D and 36 μm/D, respectively, whereas CT decreased at the rate of −14 μm/D (all *P* < 0.001) [see Table S4 in Additional file 2]. The total LT increased by up to 3.7% at maximum demand, a change primarily driven by the nucleus (NT), which exhibited substantial relative increases of up to 10.1% at 6 D. CT changes were initially small but became progressively larger and reached statistical significance at higher demands, with a 5.5% decrease at 6 D [see Table S5 in Additional file 2].

Raincloud plots in Fig. 5 illustrate that the distributions of LT and NT change were consistently shifted above zero at all accommodative demands, whereas CT distributions were displaced below zero and became increasingly negative with demand. The *P* value heatmap in Fig. S1b indicates that the LT and NT changes were uniformly colored, corresponding to *P* < 0.001, whereas CT had higher *P* values. The effect-size heatmap shows LT and NT as uniformly warm and saturated, indicating an overall thickening effect, whereas CT at higher demands is strongly cool-colored, emphasizing the opposite effect (Fig. 3b).

### Changes in OSD zones

**Fig. 6:**
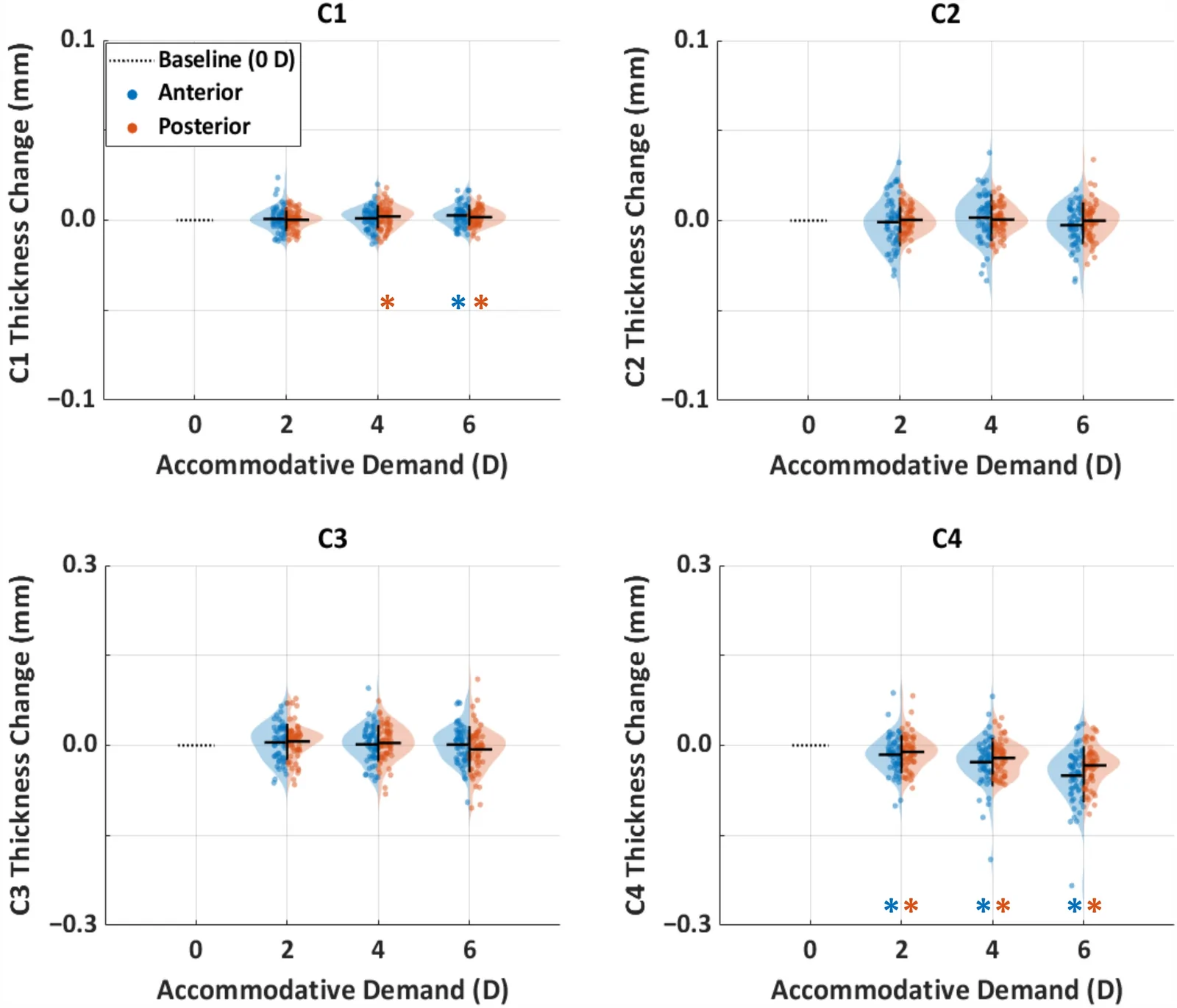
Distribution plots of accommodation-related thickness change in anterior and posterior optical signal discontinuity (OSD) zones C1–C4. Asterisk indicates statistical significance with α ≤ 0.05; Each panel shows the distribution of change from the 0 D baseline at 2 D, 4 D, and 6 D for a given zone, with anterior and posterior thickness changes displayed as two mirrored half-cloud distributions within the same panel, together with individual eyes and summary statistics for each demand

Cortical OSD zones anterior and posterior to the nucleus showed heterogeneous accommodative behavior, with robust, demand-dependent changes confined to the innermost cortical layers (Fig. 6).

Across most anterior C1–C3 and posterior C2–C3 layers, the mean thickness changes with accommodative demand were very small (≤ 5 μm) and associated with non-significant *P* values (*P* ≥ 0.13) and negligible effect sizes, indicating no meaningful accommodative deformation in those regions. The posterior C1 zone exhibited statistically significant changes at certain individual demands (4 D and 6 D), but these were of very small magnitudes with small effect sizes, consistent with weak modulation. By contrast, both the anterior and posterior C4 layers demonstrated clear, demand-dependent changes. Specifically, the anterior C4 zone thinned progressively with accommodation, at a rate of −8 μm/D (*P* < 0.001). The posterior C4 zone showed a similar monotonic decrease, at −5.52 μm/D (*P* < 0.001) [see Table S6 in Additional file 2]. When expressed as a percentage change, most peripheral intra-lenticular zones (anterior and posterior zones C1–C3) showed only modest deformation (< 3% at 6 D). In contrast, the innermost cortical zones (anterior and posterior C4) underwent significantly larger relative changes, decreasing in thickness by approximately 7%–8% at 2 D and reaching 22%–25% at 6 D [see Table S7 in Additional file 2]. These findings indicate that accommodative deformation is concentrated within the inner cortical layers, whereas the peripheral zones remain relatively stable.

Figure 6 illustrates these patterns in raincloud plots of thickness change versus accommodative demand for each cortical zone. For zone C1, both the anterior and posterior distributions remained tightly clustered around zero across all demands, with only subtle posterior shifts at 4 and 6 D. Zone C2 showed strongly overlapping anterior and posterior distributions centered near zero at 2–6 D, consistent with the minimal mean changes and non-significant *P* values. The distribution of C3 thickness was not tightly centered at zero, and all C3 thickness changes were non-significant. In contrast, the C4 panel shows anterior and posterior thickness distributions that progressively shift to more negative values with increasing demand, with high statistical significance at 2, 4, and 6 D.

Figs. S1d–e present heatmaps of the *P* values after Bonferroni correction. Anterior C1–C3 are largely represented by higher *P* value colors at all non-zero demands except for anterior C1 at 6 D, reflecting the small, non-significant changes in these layers, whereas anterior C4 forms a continuous band of low *P* values across all demands. In the posterior *P* value heatmap, posterior C2–C3 is dominated by high *P* value colors, consistent with negligible changes, while posterior C1 displays only focal islands of low *P* values at those specific demands where the table reports significance (4 D and 6 D). Posterior C4 appears as a continuous low-*P* band across non-zero demands, mirroring the highly significant thinning observed in this region. Figure 3d–e summarize the corresponding effect size heatmaps. The anterior and posterior C1 zones showed small positive values that increased modestly with demand; similarly, the posterior C3 at 2 D showed a more positive shade corresponding to its small effect. In contrast, both the anterior and posterior C4 displayed increasingly negative hues with rising demand, highlighting that the largest thinning was confined to the C4 zones.

### Change in the radii of curvature of the ocular surfaces

**Fig. 7:**
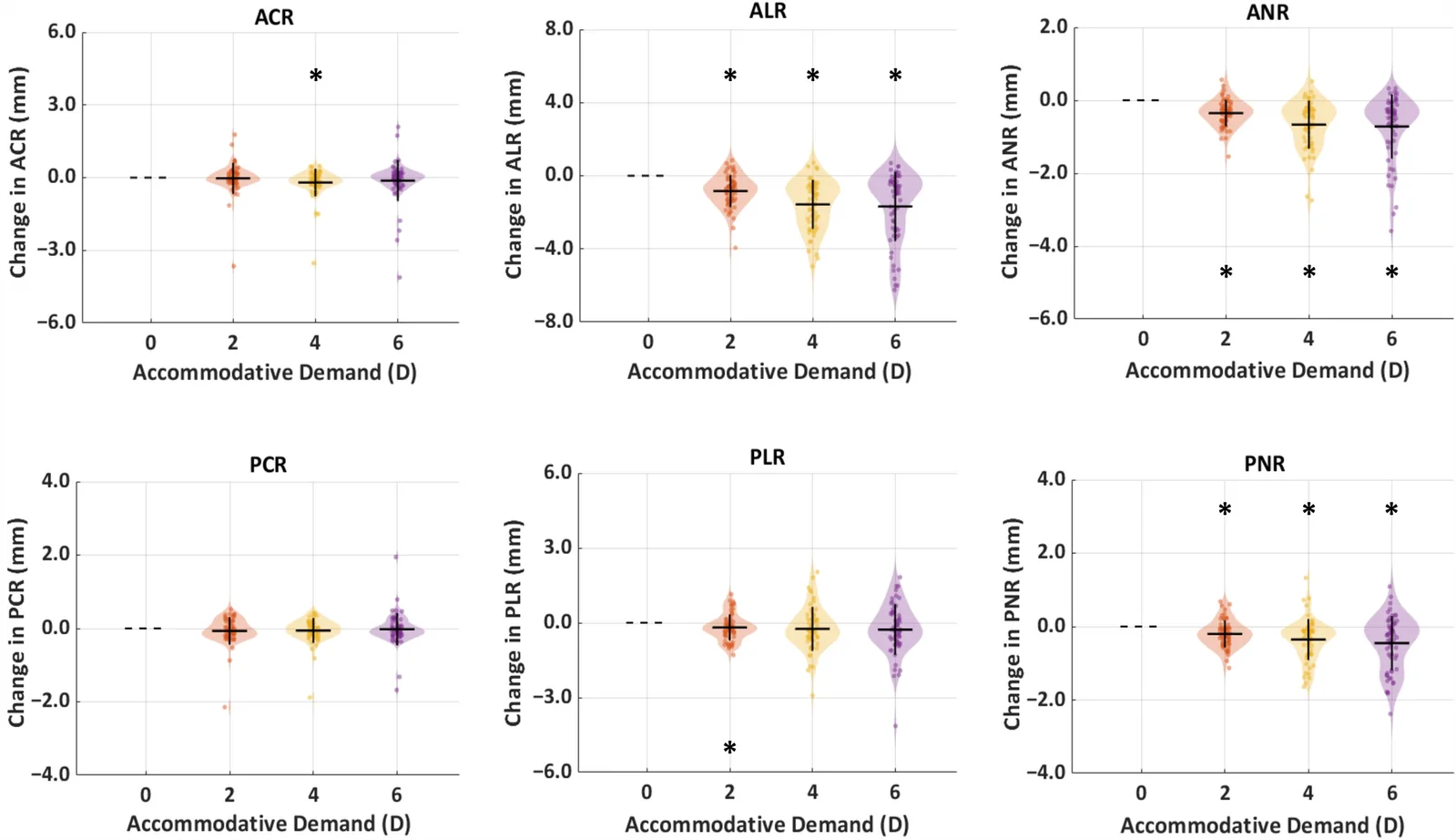
Raincloud plots of accommodation-related changes in curvatures of the ocular surfaces. Asterisk indicates statistical significance with α ≤ 0.05; Each panel shows the distribution of change from the 0 D baseline at 2 D, 4 D, and 6 D for anterior and posterior corneal lens and nuclear radius of curvature; ACR, anterior corneal radius; ALR, anterior lens radius; ANR, anterior nuclear radius; PCR, posterior corneal radius; PLR, posterior lens radius; PNR, posterior nuclear radius

Curvatures of the lens and nucleus showed pronounced demand-dependent changes during accommodation, particularly on the anterior surfaces (Fig. 7).

The anterior lens, anterior nuclear, and posterior nuclear radii of curvature (ALR, ANR, and PNR, respectively) exhibited large negative effect sizes and 95% CIs entirely below 0 (*P* < 0.001), reflecting a systematic decrease in radius with increasing demand. The PLR changed significantly at 2 D, although the magnitude of this effect weakened at higher demands and was not significant after the Bonferroni correction. In contrast, the corneal radii (ACR and PCR) showed only small and inconsistent changes across all demands. Per-diopter change analysis confirmed large negative values for ALR, ANR, and PNR (approximately −289 μm/D, −123 μm/D, and −75 μm/D, respectively; all *P* < 0.001), whereas PLR displayed only a borderline effect (−44 μm/D, *P* = 0.05) [see Table S8 in Additional file 2]. Percentage change analysis of the surface curvatures revealed that accommodative remodeling was dominated by the lens and nucleus, with minimal involvement of the cornea. While the corneal radii (ACR and PCR) remained relatively stable (≤ 2.54%), the ALR and ANR exhibited the most substantial decrease, reaching approximately 12%–13% at 6 D. Intermediate changes were observed in the PNR (up to 8%), whereas PLR changes remained modest and should be interpreted cautiously, given the lack of significance at higher demands [see Table S9 in Additional file 2].

The raincloud plots shown in Fig. 7 illustrate these patterns for the individual distributions. ALR, ANR, and PNR show progressively more negative mean changes in radius with increasing demand, accompanied by relatively tight distributions and minimal overlap with zero. The PLR displayed a similar trend but with broader distributions and smaller shifts away from zero, consistent with its non-significant change at higher demands and weaker overall contribution. In contrast, the ACR and PCR rainclouds remained centered near zero, with wide overlapping distributions and no consistent displacement across the demands. Fig. S1c and Fig. 3c summarize the Bonferroni-corrected *P* values and effect sizes, respectively. For ALR, ANR, and PNR, the effect-size heatmap showed uniformly large negative values across all accommodative demands, whereas the corresponding *P* value map was saturated at low values, underscoring the strength and consistency of these effects. PLR shows an intermediate pattern, with moderate negative effect sizes and mixed evidence across accommodative demands (*P* = 0.02 at 2 D, *P* = 0.10 at 4 D and *P* = 0.14 at 6 D), in line with its borderline per-diopter estimate. In contrast, the ACR and PCR tiles in the *P* value and effect-size heatmaps remained in the non-significant, near-zero range.

### Accommodation vs. disaccommodation

Accommodative and disaccommodative responses were similar across most of the biometric parameters. Although LT, AD, and ACD differed significantly at baseline, these mean differences were much smaller than the overall accommodative changes (Table 1). Layer-wise linear mixed-effects models showed small differences between accommodative directions and weak interactions with demand, meridian, sex, or eye.

**Table 1:** Summary of accommodation-related group and condition comparisons

Comparison	Parameter	Baseline difference (at 0 D)	Effect size	Mixed-model β (95% CI)	Mixed-model *P* values	Observation
A–D	LT	A < D	0.72	LT: 0.0345 mm [0.022, 0.046]	*P* < 0.001	Statistically significant with small effect sizes; no meaningful anatomical change
A–D	AD, ACD	A > D	0.53	AD: −0.0255 mm [−0.039, −0.012], ACD: −0.0219 mm [−0.035, −0.009]	*P* < 0.001	
H–V	ALR, ANR	H > V	0.6–0.9	ALR: −0.289 mm [−0.851, 0.273], ANR: −0.192 mm [−0.447, 0.063]	*P* > 0.05	Curvature shows mild anisotropy for posterior lens surface; effect sizes are small indicating no anatomical change
H–V	PLR	H < V	0.6	PLR: 0.490 mm [0.047, 0.934]	*P* = 0.031	
M–F	VCD, AL, PD	M > F	0.42–0.77	VCD: 0.531 mm [0.156, 0.906], AL: 0.20–0.30 mm [CI > 0], PD: 0.459 mm [−0.217, 1.135]	*P* < 0.05 PD: 0.18	True structural sex differences except for PD showing a weak baseline sex effect
M–F	ANR, PNR	M > F	0.75–0.95	ANR: 0.715 mm [0.131, 1.299], PNR: 0.634 mm [0.174, 1.094]	*P* < 0.05	
M–F	Anterior and posterior C4	M < F	0.43–0.76	Anterior C4: −0.0385 mm [−0.076, −0.001], Posterior C4: −0.0181 mm [−0.041, 0.005]	Anterior C4: 0.046, Posterior C4: 0.12	Anterior C4 zone tends to be slightly thicker in females, with small effect sizes and weak demand dependence
M–F	AL slope	M < F	0.57			Dynamic effect
OS–OD	Anterior and posterior C2	OS > OD	0.62–0.76	Anterior C2: 0.01 mm [CI around 0], Posterior C2: 0.0088 mm [−0.0022, 0.0198]	Anterior C2: 0.05−0.10; Posterior C2: 0.12	Small inter-ocular axial and cortical bias except for posterior C2
OS–OD	AL	OS < OD	0.58	−0.02 to −0.05 mm [CI < 0]	*P* < 0.001	

For each comparison the table lists the direction of the baseline difference at 0 D, for each parameter within the group, the corresponding linear mixed-effects coefficient at baseline (β and 95% CI), associated *P* value, and a qualitative observation

*ACD* = anterior chamber depth; *AD* = aqueous depth; *AL* = axial length; *ALR* = anterior lens radius; *ANR* = anterior nuclear radius; *CI* = confidence interval; *LT* = lens thickness; *PD* = pupil diameter; *PLR* = posterior lens radius; *PNR* = posterior nuclear radius; *VCD* = vitreous chamber depth. *A–D* indicates accommodation vs. disaccommodation; *H–V* indicates horizontal scan vs. vertical scan; *M–F* indicates male vs. female; *OS–OD* indicates left vs. right eyes in the population

### Accommodation across meridians

Horizontal and vertical scans revealed no significant differences in most parameters acquired in the relaxed state (baseline). The PLR at baseline showed statistically significant differences between the meridians; however, there was no consistency across the demands (Table 1). Baseline analyses of vertical–horizontal differences across layers, sex, eye, and accommodative direction showed small offsets with CIs that typically included zero. In the mixed-effects models, the curvature parameters showed only modest effects and did not exhibit a coherent pattern of orientation-related interactions across demands or groups.

### Sex and laterality effects

Several biometric parameters showed statistically significant and directionally consistent sex-related differences (Table 1). At 0 D, males had longer VCD and AL and tended to have larger PD than females, with medium to large effect sizes, and these differences remained similar across accommodative demands. In the mixed-effects models, sex showed robust main effects on VCD and AL after adjusting for eye and orientation, whereas sex-by-demand interactions in these parameters and in PD were small, indicating that the sex differences are largely demand-independent structural dimorphisms rather than accommodation-dependent effects.

Radius of curvature analyses indicated that the anterior and posterior nuclear radii were larger in males than in females, with high directional consistency across accommodative demands. Within the cortex, the anterior C4 baseline thicknesses were greater in females than in males, and the posterior C4 showed a similar but weaker trend that remained consistent across accommodative demands. Females also exhibited a slightly higher rate of axial elongation per diopter of accommodation than males; although this difference was statistically significant (*P* < 0.05), it was smaller in magnitude than the baseline AL difference at 0 D. The mixed-effects model supported the view that sex differences in AL are primarily a baseline anatomical offset, with only a modest additional sex difference in the accommodative elongation slope.

Interocular comparisons between the left (OS) and right (OD) eyes revealed only modest structural differences (Table 1). Most parameters showed non-significant OS–OD differences with small effect sizes, indicating a largely symmetric ocular anatomy. The AL was slightly but consistently shorter in OS than in OD, representing a small but robust axial bias. In addition, the anterior C2 zone was marginally thicker in OS than in OD, again reaching statistical significance despite the small magnitude of the difference. In the mixed-effects analyses, OS versus OD exerted small but consistent main effects on AL and selected cortical layers, whereas interactions with demand and orientation were weak, indicating that these interocular asymmetries were largely additive and not strongly dependent on accommodative demand or the meridian. Baseline vertical–horizontal differences were also similar between OS and OD across layers and groups, with overlapping CIs for the mean vertical–horizontal differences between the eyes.

## Discussion

This study provided a detailed quantitative understanding of accommodative changes in the eye, with particular attention to the crystalline lens. We integrated high-speed, long-range SS-OCT imaging with advanced image segmentation and precise biometry. Within this whole-eye framework, we focused on characterizing the OSD zones of the crystalline lens during accommodation. To the best of our knowledge, this aspect of lens remodeling has not been systematically investigated in relation to accommodation.

Intraocular morphology was consistent with previous findings from ultrasound biomicroscopy and other SS-OCT studies. ACD and VCD decreased with increasing accommodative stimulus, consistent with forward movement of the anterior lens surface and subtle shortening of the posterior segment during accommodation [8, 26, 27]. The CCT did not show statistically significant changes at any accommodative demand, and the observed differences were small, with small effect sizes and narrow 95% CI values close to zero. Taken together, these metrics indicated negligible corneal involvement in accommodative deformation. This interpretation is consistent with previous anterior segment OCT studies, which generally reported no significant changes in CCT across accommodative states, despite clear alterations in ACD, LT, and axial biometry [26, 28]. The small but consistent increase in AL at 2 and 4 D agrees with the findings of Drexler et al., who interpreted accommodation-induced eye elongation as a transient mechanical deformation of the posterior pole driven by ciliary muscle contraction rather than a manifestation of true scleral growth [29]. However, while several studies have reported significant increases in AL during accommodation, others have found minimal or no detectable change under similar conditions, indicating that any elongation is small and may depend on age, refractive status, and measurement sensitivity [30, 31]. PD decreased systematically with increasing accommodative demand, consistent with the pupillary component of the near triad, in which accommodation, convergence, and miosis are co-activated for near viewing [32]. Collectively, these whole-eye metrics support a model in which accommodation is expressed through anterior segment redistribution and subtle posterior deformation accompanied by strong miosis, while the corneal structure remains unchanged [28, 30, 31].

Beyond these whole-eye and anterior segment findings, the crystalline lens itself underwent marked geometric change with accommodation. Whole-lens and layer-specific analyses showed that the lens thickened linearly overall, driven primarily by nuclear thickening with compensatory cortical thinning that became statistically robust at higher demands. These parallel changes support the view that accommodation is predominantly nuclear-driven, with cortical redistribution and modulation of capsule tension shaping the observed lens deformation [4, 9, 33, 34]. Detailed zonal analysis within the lens further supports this view of accommodation as an inner cortical and nuclear phenomenon.

Across the more peripheral anterior C1–C3 and posterior C2–C3 zones, the thickness changes with increasing demand were minimal. These statistically non-significant changes, associated with negligible effect sizes, indicate that the outer cortical layers remained largely stable during accommodation. The posterior C1 zone showed only small, isolated changes at specific stimulus levels, consistent with weak secondary modulation rather than a primary role in lens shape changes. In contrast, C4 zones bordering the nucleus exhibited a clear accommodative signature. Both the anterior and posterior C4 thinned monotonically with increasing demand, with the anterior C4 showing a larger rate of change and the posterior C4 demonstrating a similar, although slightly smaller, effect. This pattern localizes accommodative remodeling to the inner cortex adjacent to the nucleus and indicates that these zones deform in a coordinated manner with nuclear thickening rather than being purely passive entities.

This selective engagement of the inner cortex is broadly consistent with mechanical models in which most accommodative shape changes are concentrated in the central lens mass, whereas the capsule and peripheral cortex primarily transmit zonular forces and enforce mechanical boundary conditions on the deforming lens [5, 33, 35, 36]. Taken together, this behavior implies that the nucleus and immediately adjacent inner cortex are the principal substrates of accommodative deformation, with more peripheral cortical layers maintaining the overall lens architecture and providing a mechanically supportive shell.

The curvature results help link the observed biometric distance changes to the underlying deformation of the crystalline lens surfaces. A large demand-dependent decrease in the anterior lens, anterior nuclear, and posterior nuclear radii of curvature, coupled with large negative effect sizes, indicates that most accommodative shape changes are achieved by coordinated steepening of the central lens complex [9, 34]. The posterior lenticular radius decreased with accommodation but reached statistical significance only at low demand. Together with its small effect sizes and borderline per-diopter slope, this pattern suggests that it makes only a modest secondary contribution to the overall deformation. In contrast, the minimal or inconsistent changes in the anterior and posterior corneal radii across all demands confirm that the corneal shape is largely decoupled from accommodative effort in healthy eyes and that the observed biometric changes are predominantly lens-driven [37]. Considered together with the intraocular distance and OSD-zonal thickness data, these curvature findings support a coherent picture of accommodation as a central lens phenomenon with limited peripheral or corneal participation [8, 26, 38].

Group comparisons across accommodation direction, meridian, sex, and eye laterality provided additional context for the robustness and generalizability of the observed accommodative patterns. Bidirectional testing also revealed negligible hysteresis between accommodation and disaccommodation with similar structural changes in both directions, suggesting a limited viscoelastic delay or plastic deformation within the physiological accommodation range studied. Previously published studies comparing accommodation and disaccommodation have focused on dynamic response properties (latency, peak velocity, and lag) [39] rather than on direction-dependent differences in steady-state ocular biometry and reported hysteresis mainly as a temporal phenomenon. In line with this, our quasi-static measurements showed no large systematic differences or meaningful correlations between accommodative and disaccommodative structural changes, implying that any hysteresis effects described in dynamic studies are either primarily temporal or too small to produce detectable asymmetry in the steady-state biometric parameters under the present testing conditions. This conclusion was reinforced by the mixed-effects models, which showed only small main effects and weak interactions of accommodative direction on layer-wise structural changes when repeated measures and covariates were considered.

The absence of large, systematic differences between the horizontal and vertical meridians in the key lens and nuclear curvature metrics within the central 3-mm diameter zone indicates that the accommodative deformation of the lens is approximately axisymmetric, with similar radii of curvature in orthogonal directions. This interpretation is consistent with multi-radial Scheimpflug studies that directly compared the anterior lens curvature along the horizontal and vertical meridians, reporting broadly comparable radii within the central pupil zones [40]. Our OCT-derived nuclear curvature data extend this evidence of meridional symmetry from the anterior surface to the lens interior by showing that no strong horizontal-vertical curvature differences are present in the central nucleus of eyes with low astigmatism. These findings support the common modeling assumption that the lens nucleus can be treated as rotationally symmetric around the optical axis, as adopted in Scheimpflug-based nucleus shape and volume analyses [34] and are further supported by mixed-effects curvature analyses, which did not reveal consistent orientation-related interactions across accommodative demands.

The sex-based differences in our cohort were in line with broader evidence that healthy male and female eyes differ systematically in several biometric characteristics [41]. In particular, males showed longer VCD, and greater AL, with medium to large effect sizes, and these offsets remained essentially constant across accommodative demands, consistent with stable anatomical dimorphisms rather than accommodation-dependent effects [42, 43]. This pattern was confirmed in the mixed-effects models, in which sex had strong effects on VCD and AL and modest effects on PD, but only small sex-by-demand interactions. Beyond these established dimorphisms, our data revealed that females in the cohort exhibited a slightly higher rate of axial elongation per-diopter of accommodation than males, a thicker anterior C4 zone, a similar but weaker trend for the posterior C4 cortical zone, and smaller anterior and posterior nuclear radii of curvature across demands. In the mixed-effects analysis, these findings corresponded to modest but significant sex-by-demand effects on AL and robust sex main effects on C4 thickness and nuclear curvature after adjusting for the eye and meridian. To our knowledge, sex differences in accommodative AL change, inner cortical (C4) thickness, and nuclear curvature have not been reported previously and thus appear to represent novel, subtle structural dimorphisms of the accommodating lens that warrant further investigation.

Additionally, interocular (OD–OS) differences were minimal for most parameters, with small mean offsets and effect sizes, indicating a largely symmetric ocular anatomy. The AL was slightly but consistently shorter in OS than in OD, and the anterior C2 zone was marginally thicker in OS than in OD; however, these differences were small in magnitude and within the range reported for normal interocular variation. This pattern of high bilateral symmetry across the axial, anterior segment, and curvature metrics supports the interpretation that the observed changes reflect intrinsic ocular morphology, rather than measurement artifacts or eye-specific biases [44]. Baseline vertical–horizontal differences were also similar between OS and OD, with overlapping CIs for mean vertical–horizontal differences across layers and groups, further supporting bilateral meridional symmetry in this cohort.

Some limitations should be considered when interpreting these results. First, the study sample comprised relatively healthy young adults with low refractive error and low astigmatism, and eyes with a spherical equivalent beyond ±3.50 D were excluded. These results, therefore, may not generalize directly to highly myopic, hyperopic, or highly astigmatic eyes, in which accommodative behavior and axial dynamics can differ [45, 46]. One eye had a history of corneal refractive surgery, which should be considered when considering corneal and anterior segment metrics; however, excluding this eye did not materially alter the main outcomes. Second, although the sample size (60 eyes) is moderate, all primary analyses are supported by effect sizes and narrow CIs, suggesting that the study was adequately powered to detect small to medium within-subject accommodative effects; nevertheless, subtle subgroup interactions (e.g., sex, meridian) may require larger cohorts to confirm. Third, the reported radii of curvature are derived from best-fit spherical measurements over the central 3-mm diameter zone. However, this may not fully capture aspheric or complex lens-shape changes, particularly at higher accommodative demands. Fourth, the current refraction correction pipeline relies on theoretical phase refractive indices extrapolated from the chromatic-dispersion models proposed by Atchison and Smith [25], rather than standard group refractive indices. Mathematically, our utilization of phase indices introduces a systematic thickness overestimation of approximately 0.65%–0.72% across ocular media. Because this error operates as a uniform scaling factor across all accommodative states, it did not alter the relative structural deformations or statistical trends reported in this cohort. Nonetheless, recent advancements by Tian et al. established highly accurate and empirically measured ocular group refractive indices [47]. Future studies tracking absolute ocular biometry should adopt these updated empirical values to overcome these limitations and maximize true geometric precision. The eye model adopted in this study simplified the GRIN distribution within the lens by implementing a double-shell (cortex and nucleus) model of the crystalline lens. Finally, posterior lenticular curvature estimates should be interpreted more cautiously than anterior estimates because their reconstruction is more sensitive to assumptions regarding lens refractive index distribution and GRIN, particularly at the back surface [48]. Simplified GRIN representations are known to provide reasonable estimates of the central power and curvature in healthy lenses, but they do not fully capture regional index variations; therefore, detailed posterior shape parameters are likely to be less precise than the corresponding anterior and nuclear metrics.

## Conclusions

This study demonstrated that accommodation in healthy young adults is driven mainly by central lens remodeling. Nuclear thickening and selective thinning of the adjacent inner cortex (C4) accounted for most shape changes, while the peripheral cortex remained largely stable. The OSD-based zonal analysis offers a novel, depth-resolved view, indicating that the pre-existing discontinuity architecture acts as a layered deformation framework rather than a static anatomical feature. Curvature analyses revealed clear steepening of the anterior lens and nuclear surfaces with negligible changes in corneal geometry, supporting the idea that accommodation is a central lens phenomenon in eyes with low ametropia. Subtle sex-related differences in the inner CT, nuclear curvature, and accommodative axial elongation suggest previously unrecognized structural dimorphisms relevant to individualized biomechanical models. These results improve our understanding of normal accommodative function and establish a quantitative baseline for lens zone deformation and whole-eye biometric responses that can support future studies on presbyopia and accommodative dysfunction.

## Supplementary Information

Additional file 1. Within-session repeatability and inter-session reproducibility of OSD zone thickness measurements.

Additional file 2. Statistical significance of changes in biometric parameters of the eye with respect to baseline (no accommodative demand). Absolute and percentage changes in eye biometry relative to the 0 D baseline.

## Data Availability

The datasets generated and analyzed during the current study are available in the Repository for Open Data, https://doi.org/10.18150/SAH7WP.

## Abbreviations

ACR: Anterior corneal radius
ACD: Anterior chamber depth
AL: Axial length
ALR: Anterior lens radius
ANR: Anterior nuclear radius
CCT: Central corneal thickness
CI: Confidence interval
CT: Cortical thickness
GRIN: Gradient refractive index
IoU: Intersection over union
LT: Lens thickness
NT: Nuclear thickness
OCT: Optical coherence tomography
OSD: Optical signal discontinuity
PCR: Posterior corneal radius
PD: Pupil diameter
PLR: Posterior lens radius
PNR: Posterior nuclear radius
SS-OCT: Swept-source optical coherence tomography
VCD: Vitreous chamber depth
