# Supplementary material for: Layer-specific quantitative analysis of accommodation in the human lens using long-range OCT and machine learning
Source: Eye Vis (Lond). 2026 Nov 1;13:47. doi: 10.1186/s40662-026-00517-w (PMC13723935; doi:10.1186/s40662-026-00517-w)
Supplement: Supplementary file 1 [file 40662_2026_517_MOESM1_ESM.pdf]

Additional file 1

Within-session repeatability and inter-session reproducibility of the thickness measurement of the optical signal discontinuity (OSD) zones

Manually delineated OSD zones were extracted from densitometric profiles (optical coherence tomography [OCT] B-scans) by a single trained observer using a standardized protocol. The reliability of the resulting measurements was evaluated in the unaccommodated (0 D) and accommodated (6 D) states. For repeatability, eight subjects underwent three repeated measurements in a single session and the OSD zones were segmented for each measurement. For reproducibility, three subjects underwent measurements on three separate days, each including three repeated measurements with manual segmentation. The coefficient of variation (CV) was defined as the ratio of the standard deviation to the mean and was expressed as a percentage. The intraclass correlation coefficient (ICC; two-way mixed, single measure, and absolute agreement) was also calculated for each zone and condition from repeated measurements.

The reported metrics capture the combined effects of acquisition variability, biological variability, and the manual segmentation step, and thus quantify intra-observer reliability, but do not isolate segmentation reproducibility on identical images or inter-observer agreement.

The results are presented in Table S1. For the peripheral OSD zones (anterior and posterior C1), intra-observer repeatability and inter-session reproducibility were excellent, with CV values typically around 2%–5% and ICCs close to 0.90−0.99 at both 0 D and 6 D, indicating highly stable manual OSD measurements. Intermediate OSD zones (anterior and posterior C2 and C3) generally showed good to excellent reliability, with ICCs mostly above 0.85 and CVs in the 1%–11% range, supporting robust manual segmentation in these regions, apart from isolated conditions with lower ICC (posterior C2 at 0 D). For the inner OSD zones (anterior and posterior C4), reliability was lower, especially at 6 D, with CVs in the 11%–20% range and ICCs dropping below 0.5 for some conditions, likely reflecting a combination of increased anatomical variability

and reduced local signal-to-noise rather than solely segmentation inconsistency.

Table S1. Within-session repeatability and inter-session reproducibility of optical signal discontinuity (OSD) zones based on manual segmentation at unaccommodated (0 D) and accommodated (6 D) states. Higher intraclass correlation coefficient (ICC) and lower coefficient of variation (CV) indicate better repeatability and reproducibility.

**Repeatability**							
Zone		0 D			6 D		
		CV	ICC	*P* value	CV	ICC	*P* value
Anterior	C1	2.99	0.94*	< 0.001	2.16	0.98*	< 0.001
	C2	10.72	0.86*	< 0.001	8.41	0.91*	< 0.001
	C3	4.19	0.85*	< 0.001	4.21	0.94*	< 0.001
	C4	8.12	0.76*	< 0.001	10.57	0.62*	0.003
Posterior	C1	5.19	0.79*	< 0.001	3.90	0.93*	< 0.001
	C2	16.57	0.34	0.070	9.65	0.79*	< 0.001
	C3	4.47	0.87*	< 0.001	5.04	0.90*	< 0.001
	C4	11.65	0.58*	0.004	6.72	0.86*	< 0.001
**Reproducibility**							
Zone		0 D			6 D		
		CV	ICC	*P* value	CV	ICC	*P* value
Anterior	C1	1.82	0.99*	< 0.001	2.02	0.99*	< 0.001
	C2	4.55	0.97*	< 0.001	10.75	0.89*	0.009
	C3	1.54	0.95*	< 0.001	4.35	0.72*	0.047
	C4	8.07	0.84*	0.003	13.34	0.28	0.187
Posterior	C1	3.00	0.91*	0.001	5.06	0.77*	0.004
	C2	6.47	0.84*	0.001	4.88	0.96*	< 0.001
	C3	1.48	0.97*	< 0.001	4.87	0.63	0.070
	C4	11.55	0.39	0.148	20.34	0.002	0.442

CV = coefficient of variation; ICC = intraclass correlation coefficient; C1–C4 indicate anterior/posterior cortical OSD zones; Asterisk indicates statistical significance with $\alpha \leq 0.05$.
