# Supplementary material for: Layer-specific quantitative analysis of accommodation in the human lens using long-range OCT and machine learning
Source: Eye Vis (Lond). 2026 Nov 1;13:47. doi: 10.1186/s40662-026-00517-w (PMC13723935; doi:10.1186/s40662-026-00517-w)
Supplement: Supplementary file 2 [file 40662_2026_517_MOESM2_ESM.pdf]

Additional file 2

Statistical significance of the changes in biometric parameters of the eye with respect to the baseline (no accommodation demand). Absolute and percentage change in eye biometry relative to the 0 D baseline.

The file shows heatmaps displaying Bonferroni-corrected *P* values across the demands for each biometric parameter (Fig. S1).

Additionally, this file provides detailed numerical values, as well as the percentage change in accommodation-induced changes in ocular biometry relative to the 0 D baseline. The absolute accommodative changes in several parameters were very small in terms of physical units (Tables S2, S4, S6, and S8). To facilitate interpretation of these subtle effects, we therefore re-expressed the same data as percentage changes from baseline. Percentage changes from baseline, calculated as $(|(X_D - X_{0D})|/X_{0D}) \times 100$ (Tables S3, S5, S7 and S9).

Fig. S1. Heatmaps of *P* values for accommodation-induced changes in parameters. **a** Intraocular distances and pupil diameter across accommodative demands; **b** Thickness of the crystalline lens and its anatomical components; **c** Radii of curvature of the ocular surfaces; **d** Thicknesses of anterior cortex OSD zones C1–C4; **e** Thicknesses of posterior cortex OSD zones C1–C4. ACD, anterior chamber depth; ACR, anterior corneal radius; AD, aqueous depth; AL, axial length; ALR, anterior lens radius; ANR, anterior nuclear radius; CCT, central corneal thickness; CT, cortical thickness; LT, lens thickness; NT, nuclear thickness; PCR, posterior corneal radius; PD, pupil diameter; PLR, posterior lens radius; PNR, posterior nuclear radius; VCD, vitreous chamber depth.

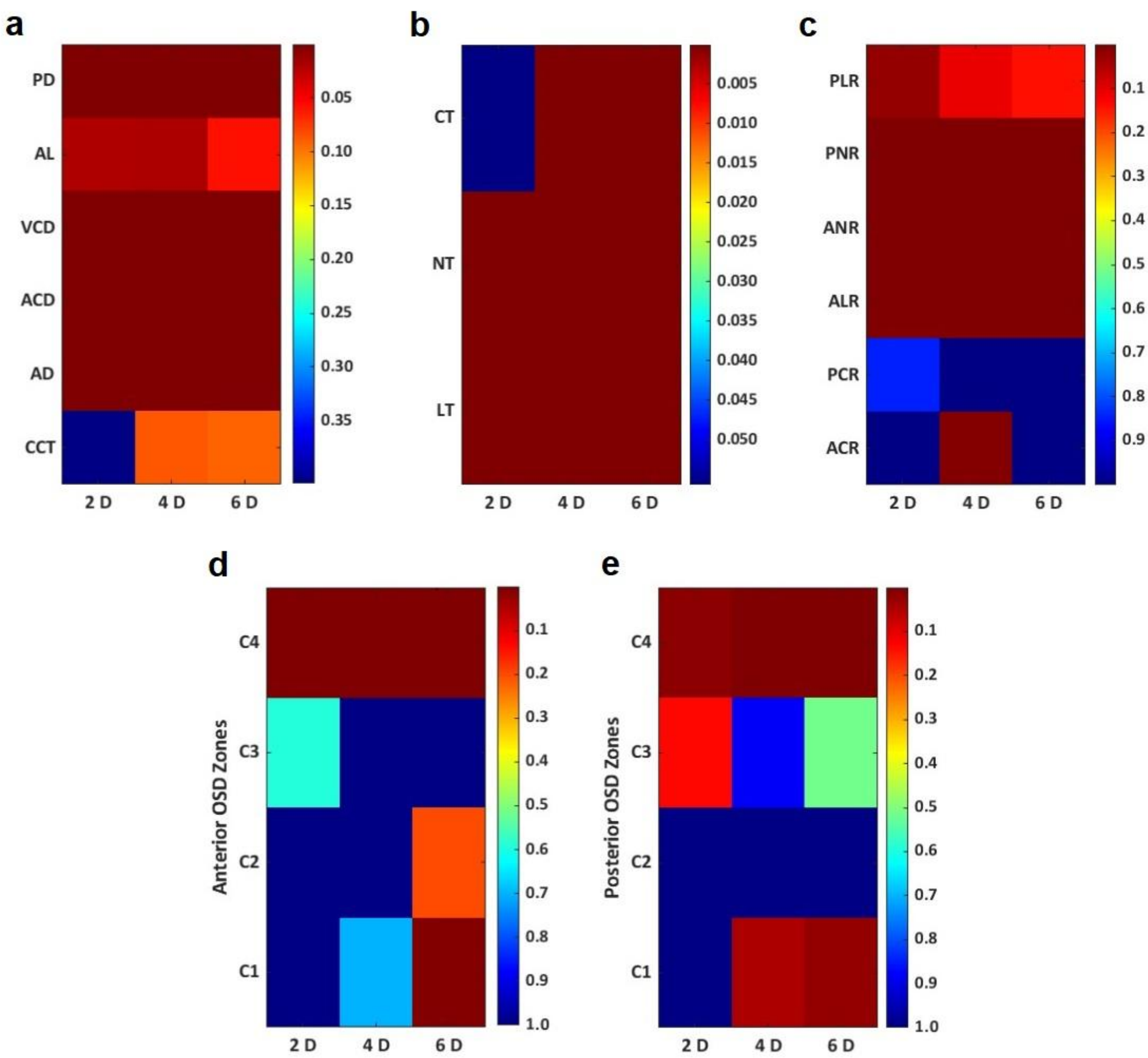

Changes in the intraocular distances and pupil diameter

Table S2. Change in intraocular distances and pupil diameter at each accommodative demand. Values are relative to the 0 D baseline. Columns report the mean change (mm) at 2, 4, and 6 D, and the change per-diopter (μm/D).

Parameter	Change at			Change per diopter (μm/D)
	2 D (mm)	4 D (mm)	6 D (mm)	
CCT	0.002 ± 0.010	0.002 ± 0.009	0.002 ± 0.014	0.31*
AD	−0.052 ± 0.037*	−0.092 ± 0.071*	−0.097 ± 0.091*	−16.58*
ACD	−0.050 ± 0.039*	−0.090 ± 0.070*	−0.095 ± 0.090*	−16.27*

VCD	−0.014 ± 0.020*	−0.029 ± 0.035*	−0.038 ± 0.044*	−6.46*
AL	0.006 ± 0.016*	0.007 ± 0.019*	0.007 ± 0.028	1.12*
PD	−0.185 ± 0.239*	−0.403 ± 0.513*	−0.472 ± 0.794*	−81.66*

ACD = anterior chamber depth; AD = aqueous depth; AL = axial length; CCT = central corneal thickness; PD = pupil diameter; VCD = vitreous chamber depth; Asterisk indicates statistical significance with α ≤ 0.05; corresponding *P* values are shown in Fig. S1a (heatmaps).

Table S3. Percentage differences in ocular biometry parameters across accommodative demands. This table reports group-mean percentage changes at 2, 4, and 6 D relative to the group-mean baseline (0 D) for central corneal thickness (CCT), aqueous depth (AD), anterior chamber depth (ACD), vitreous chamber depth (VCD), axial length (AL), and pupil diameter (PD).

Parameter	Percentage difference with respect to		
	2 D	4 D	6 D
CCT	0.33	0.35	0.33
AD	1.69	3.00	3.16
ACD	1.37	2.47	2.61
VCD	0.08	0.17	0.23
AL	0.02	0.03	0.03
PD	2.88	6.27	7.33

The percentage change analysis confirmed that the absolute accommodative shifts in the global biometry parameters were relatively small. The central corneal thickness (CCT), vitreous chamber depth (VCD), and axial length (AL) changed by less than 1% over the full demand range. Aqueous depth (AD) and anterior chamber depth (ACD) showed modest but consistent reductions in the order of 1.7%–3.2% and 1.4%–2.6% by 6 D, respectively. Pupil diameter (PD) displayed the largest percentage change among the biometric parameters, decreasing by approximately 2.9%, 6.3%, and 7.3% at 2, 4, and 6 D, respectively. Together, these results demonstrate that aside from pupil diameter and modest anterior segment changes, accommodative effects on bulk ocular biometry remain small when expressed as percentage differences from baseline.

Changes in the thickness of the lens and its main components

Table S4. Changes in crystalline lens and component thickness at each accommodative demand. Values are relative to the 0 D baseline. Columns report the mean change (mm) at 2, 4, and 6 D, and the change per diopter (μm/D).

Parameter	Change at			Change per diopter (μm/D)
	2 D (mm)	4 D (mm)	6 D (mm)	
LT	0.071 ± 0.048*	0.126 ± 0.092*	0.142 ± 0.126*	24.03*
NT	0.082 ± 0.051*	0.159 ± 0.078*	0.215 ± 0.115*	36.13*
CT	−0.015 ± 0.047	−0.038 ± 0.065*	−0.090 ± 0.083*	−14.38*

CT = cortical thickness; LT = total lens thickness; NT = nuclear thickness; Asterisk indicates statistical significance with α ≤ 0.05; corresponding *P* values are shown in Fig. S1b (heatmaps).

Table S5. Percentage differences in thickness of the lens and its components, across accommodative demands. This table reports group-mean percentage changes at 2, 4, and 6 D relative to the group-mean baseline (0 D) for lens thickness (LT), nuclear thickness (NT) and cortical thickness (CT).

Parameter	Percentage difference with respect to 0 D at		
	2 D	4 D	6 D
LT	1.85	3.29	3.71
NT	3.82	7.43	10.08
CT	0.92	2.38	5.46

The total lens thickness (LT) increased by approximately 3.7% at the highest demand. The

nucleus (NT) showed larger relative changes, with percentage increases of approximately 3.8%, 7.4%, and 10.1% at 2, 4, and 6 D. In contrast, the cortex (CT) exhibited smaller early changes that became more substantial at higher demands, reaching 5.5% at 6 D. Together, these data confirmed that accommodative lens thickening was driven primarily by the nucleus, with a secondary but increasingly important cortical component at higher accommodative levels.

Changes in OSD zones

Table S6. Changes in anterior and posterior optical signal discontinuity (OSD) zone thickness at each accommodative demand. Values are relative to the 0 D baseline. Columns report the mean change (mm) at 2, 4, and 6 D, and the change per diopter (μm/D).

Zone		Thickness change at			Change per diopter (μm/D)
		2 D (mm)	4 D (mm)	6 D (mm)	
Anterior	C1	0.001 ± 0.007	0.001 ± 0.006	0.003 ± 0.006*	0.41*
	C2	−0.001 ± 0.014	0.002 ± 0.013	−0.003 ± 0.011	−0.26
	C3	0.005 ± 0.030	0.002 ± 0.030	0.001 ± 0.031	0.01
	C4	−0.016 ± 0.031*	−0.028 ± 0.040*	−0.050 ± 0.045*	−8.16*
Posterior	C1	0.0002 ± 0.005	0.002 ± 0.006*	0.002 ± 0.005*	0.34*
	C2	0.0004 ± 0.007	0.001 ± 0.010	−0.0001 ± 0.010	−0.01
	C3	0.007 ± 0.030	0.004 ± 0.030	−0.007 ± 0.038	−1.14
	C4	−0.011 ± 0.028*	−0.021 ± 0.027*	−0.033 ± 0.032*	−5.52*

Asterisk indicates statistical significance with α ≤ 0.05; corresponding *P* values are shown in Figs. S1d–e (heatmaps).

Table S7. Percentage differences in intra-lenticular optical signal discontinuity (OSD) zones across accommodative demands. This table reports group-mean percentage changes at 2, 4, and 6 D relative to the group-mean baseline (0 D) for the anterior and posterior cortical OSD

zones C1–C4.

Zone		Percentage thickness difference with respect to 0 D at		
		2 D	4 D	6 D
Anterior	C1	0.51	0.72	1.95
	C2	0.89	1.59	2.58
	C3	1.06	0.35	0.25
	C4	7.67	13.72	24.73
Posterior	C1	0.24	2.49	2.01
	C2	0.6	0.75	0.15
	C3	1.71	1.06	1.76
	C4	7.18	13.95	22.19

When expressed as a percentage change from baseline, most peripheral intra-lenticular zones showed only modest deformations across accommodations. Anterior and posterior C1 to C3 zone thicknesses exhibited percentage changes generally below 3% at 6 D, indicating a relatively small change in these regions. In contrast, the innermost cortical zones A-C4 and P-C4 showed larger relative changes, with values decreasing by approximately 7%–8% at 2 D and 22%–25% at 6 D. These findings support the observation that accommodative deformation is concentrated in the inner cortical layers, whereas the peripheral zones undergo comparatively minor percentage changes.

Changes in the radii of curvature of the ocular surfaces

Table S8. Changes in radii of curvature of the ocular surfaces at each accommodative demand. Values are relative to the 0 D baseline. Columns report the mean change (mm) at 2, 4, and 6 D, and the change per diopter (μm/D).

Parameter	Change at			Change per diopter (μm/D)]
	2 D (mm)	4 D (mm)	6 D (mm)	
ACR	−0.028 ± 0.643	−0.203 ± 0.573*	−0.124 ± 0.849	−27.31

PCR	−0.067 ± 0.374	−0.058 ± 0.337	−0.021 ± 0.444	−2.65
ALR	−0.838 ± 0.879*	−1.567 ± 1.341*	−1.680 ± 1.896*	−288.53*
ANR	−0.353 ± 0.366*	−0.666 ± 0.658*	−0.718 ± 0.881*	−123.42*
PNR	−0.201 ± 0.368*	−0.354 ± 0.561*	−0.451 ± 0.736*	−75.36*
PLR	−0.188 ± 0.519*	−0.244 ± 0.882	−0.275 ± 1.013	−44.12*

ACR = anterior corneal radius; ALR = anterior lens radius; ANR = anterior nuclear radius; PCR = posterior corneal radius; PLR = posterior lens radius; PNR = posterior nuclear radius; Asterisk indicates statistical significance with α ≤ 0.05; corresponding *P* values and effect sizes are shown in Fig. S1c.

Table S9. Percentage differences in radii of curvature of the ocular surfaces across accommodative demands. This table reports group-mean percentage changes at 2, 4, and 6 D relative to the group-mean baseline (0 D) for anterior and posterior corneal (ACR, PCR), lenticular (ALR, PLR), and nuclear (ANR, PNR) surfaces.

Parameter	Percentage difference with respect to 0 D at		
	2 D	4 D	6 D
ACR	0.35	2.54	1.55
PCR	0.91	0.78	0.28
ALR	6.17	11.54	12.38
ANR	6.24	11.79	12.71
PNR	3.60	6.34	8.08
PLR	1.71	2.22	2.51

To complement the absolute analyses, we also quantified the magnitude of percentage changes in the anterior and posterior radii of curvature relative to baseline. The anterior corneal radius (ACR) showed small changes, with values approximating 1.6% at 6 D, whereas PCR changed by less than 1% at all demands, indicating a negligible change during accommodation. In contrast, anterior corneal radius (ALR) and anterior nuclear radius (ANR) exhibited the largest changes, with a decrease of approximately 6% at 2 D to approximately 12%–13% at 6 D. The posterior nuclear radius (PNR) showed intermediate changes of 3.6%, 6.3%, and 8.1% at 2, 4,

and 6 D, respectively, whereas the posterior lens radius (PLR) changed modestly, on the order of 1.7%–2.5% across the examined demands. Taken together, these magnitudes indicate that accommodative remodeling is dominated by relatively large changes in nuclear and lenticular curvatures, with much smaller changes in corneal curvatures.
